# Developing and Evaluating a School-Based Tobacco and E-Cigarette Prevention Program for Deaf and Hard-of-Hearing Youth

**DOI:** 10.1177/15248399221151180

**Published:** 2023-02-09

**Authors:** Alison K. Herrmann, Burton Cowgill, Debra Guthmann, Jessica Richardson, L. Cindy Chang, Catherine M. Crespi, Everett Glenn, Michael McKee, Barbara Berman

**Affiliations:** 1 UCLA Kaiser Permanente Center for Health Equity, Los Angeles, CA, USA; 2 UCLA Fielding School of Public Health, Los Angeles, CA, USA; 3 UCLA Jonsson Comprehensive Cancer Center, Los Angeles, CA, USA; 4 Education Consultant, Auburn, CA, USA; 5 University of Michigan, Ann Arbor, MI, USA

**Keywords:** tobacco prevention and control, tobacco prevention programming, Deaf and Hard-of-Hearing, American Sign Language, e-cigarette prevention programming

## Abstract

School-based programs are an important tobacco prevention tool. Yet, existing programs are not suitable for Deaf and Hard-of-Hearing (DHH) youth. Moreover, little research has examined the use of the full range of tobacco products and related knowledge in this group. To address this gap and inform development of a school-based tobacco prevention program for this population, we conducted a pilot study among DHH middle school (MS) and high school (HS) students attending Schools for the Deaf and mainstream schools in California (n = 114). American Sign Language (ASL) administered surveys, before and after receipt of a draft curriculum delivered by health or physical education teachers, assessed product use and tobacco knowledge. Thirty-five percent of students reported exposure to tobacco products at home, including cigarettes (19%) and e-cigarettes (15%). Tobacco knowledge at baseline was limited; 35% of students knew e-cigarettes contain nicotine, and 56% were aware vaping is prohibited on school grounds. Current product use was reported by 16% of students, most commonly e-cigarettes (12%) and cigarettes (10%); overall, 7% of students reported dual use. Use was greater among HS versus MS students. Changes in student knowledge following program delivery included increased understanding of harmful chemicals in tobacco products, including nicotine in e-cigarettes. Post-program debriefings with teachers yielded specific recommendations for modifications to better meet the educational needs of DHH students. Findings based on student and teacher feedback will guide curriculum development and inform next steps in our program of research aimed to prevent tobacco use in this vulnerable and heretofore understudied population group.

Tobacco use, a leading cause of preventable death in the United States, most frequently begins in adolescence (Dierker et al., 2012). School-based programs have a recognized place in tobacco prevention efforts (U.S. Department of Health and Human Services [DHHS], 2012) with programming increasingly focused on e-cigarettes/vaping, now the most widely used tobacco product among youth (Park-Lee et al., 2021). Yet, such health programs are inaccessible to Deaf and Hard-of-Hearing (DHH) youth who use American Sign Language (ASL), the dominant language used by Deaf individuals. ASL is a visual/spatial/manual language with its own vocabulary, grammar, morphology, and syntax. It has no written version or direct translation to English. For many DHH people, English is a second or third language and lip reading is not viable or appropriate. Average English reading comprehension for Deaf ASL users is at or below a sixth-grade level (McKee et al., 2015; Zazove et al., 2013).

Tailored tobacco prevention and other health materials for DHH youth are rarely available (Smith & Samar, 2016) and health risk behavior data needed to shape programming is lacking, leaving educators to utilize and adapt approaches not culturally and linguistically tailored to their students’ needs. This gap in prevention education is concerning given issues related to self-esteem and social acceptance associated with health risk behaviors among youth are exacerbated in the DHH population (Brice & Strauss, 2016; Coll et al., 2009) and that the DHH population experiences significant barriers to receipt of health services and information (Scheier, 2009; Smith & Samar, 2016). Moreover, health-related public service announcements and educational videos largely remain inaccessible, either not captioned or available in ASL; signed health information on the internet is limited; and interpreter services in clinical settings are frequently inadequate (Kushalnagar et al., 2015; McKee et al., 2011; McKee & Paasche-Orlow, 2012; Scheier, 2009). These challenges to receipt of health information underscore the importance of school-based prevention education, including e-cigarette and tobacco use, for DHH youth.

As a result, over two decades ago, at the urging of Deaf community leaders, we initiated community-academic partnered research aimed at understanding tobacco use and addressing school-based tobacco prevention programming needs of DHH youth. Collaborating with students and educators at Schools for the Deaf in three states, we developed and assessed a tailored school-based tobacco use prevention curriculum, *Hands Off Tobacco!* (Berman, Guthmann, Crespi, & Liu, 2011). More recently, given the emergence of e-cigarettes and changes in use of social media and other communication forms, we identified a need to modify this original curriculum and obtained pilot funding to support this effort.

Reflecting present educational patterns, we included research partners from Schools for the Deaf and mainstream schools that serve DHH youth throughout the research process. Schools for the Deaf serve DHH students exclusively, use ASL as the primary mode of communication, serve youth from infancy through Grade 12, and provide on-campus housing for some students. In mainstream settings, DHH students use a variety of communication modalities, may participate in self-contained classes and/or in classes that include hearing students, often utilizing interpreters and other accommodations. In prior work, we found higher rates of tobacco use among DHH youth in mainstream schools (Berman et al., 2007), but little is known regarding current student practices.

The first step in our pilot research was to solicit information from current DHH middle school (MS) and high school (HS) students and faculty who serve these youth using qualitative methods aimed to understand students’ tobacco knowledge and use, best educational practices for DHH youth, and school programming and policies. Results of this work are described elsewhere (Cowgill et al., 2020). Guided by this information, our next step was to develop an updated, draft curriculum, *Hands Off* Tobacco *and E-Cigarettes!*, and to implement this program in participating schools. In addition, we developed a survey instrument to use in preliminary assessment of the curriculum’s effectiveness and to gain more detailed information related to DHH students’ tobacco-related knowledge, exposure, and use/intention to use tobacco products.

We sought to examine DHH students’ tobacco-related knowledge, behaviors and experiences overall, changes that occurred in relation to receipt of our tobacco prevention curriculum, and possible differences between students educated in mainstream schools and Schools for the Deaf. We hypothesized that students in both settings would experience increased knowledge and decreased intention to use tobacco products following program participation, and that given their exposure to hearing students, mainstream school students would have greater familiarity and experience with e-cigarettes. We also sought to obtain student and faculty feedback regarding the revised curriculum to guide further program modifications and next steps.

## Method

An Advisory Committee of 10 experts in DHH culture, health, and education, and a panel of eight ninth-grade “Youth Champions” from the California School for the Deaf Fremont guided the research. Participants were recruited from California’s two Schools for the Deaf (Fremont and Riverside) and two mainstream programs serving DHH students in California (San Diego Unified School District and Orange County Department of Education). Through an iterative process, both groups reviewed and provided feedback on multiple drafts of the curriculum and survey, including recommendations for the most appropriate signs to use when conveying content. The Advisory Committee also provided guidance related to the development of teacher training materials and protocols. Youth Champions provided insight into students' perceptions of tobacco products and use, experiences with marketing, and with peer pressure. In addition, these young people gave suggestions for interactive classroom activities and ways to engage students with the curriculum. Both groups helped to guide interpretation of study results, including survey findings and feedback from students and teachers. The input provided by the Advisory Committee and Youth Champions is also helping to shape the next phases of this program of research. The study was approved by the UCLA Office of Human Subjects Research.

### Conceptual Framework

We nested the revised curriculum within a broad social influences/life-skills approach, featuring Social Influences Model of Prevention components (Botvin & Griffin, 2001; Griffin & Botvin, 2010; Sussman & Cowgill, 2016). This model has been used successfully in the prevention of cigarettes, cigars, and smokeless tobacco for decades (Brannon et al., 1989; Dent et al., 1995; Levia et al., 2018; Nakkash et al., 2018; Sussman et al., 1993). It is based on enduring general principles of social behavior that apply to e-cigarettes, as well as other substances (Sussman et al., 1999; Thomas et al., 2015). The model specifies that two categories of social influences increase the risk of substance use experimentation. *Normative social influence* refers to pressure applied, explicitly or implicitly, by the peer group to make adolescents conform to group norms. Adolescents who conform to group norms receive social acceptance, and those who do not conform experience social rejection. *Informational social influence* refers to more covert pressure to make adolescents adopt attitudes and beliefs that are consistent with substance use. Information from peers, parents and siblings, tobacco advertising, or the media can make adolescents believe that tobacco use will give them a desired social image (e.g., mature, cool, sexually attractive, rebellious). Both normative and informational social influences affect middle-school-age students and encourage these young people to use e-cigarettes and tobacco. In revising the curriculum, we also retained elements consistent with social learning theory, problem-solving behavior theory, the health belief model, manipulation of normative expectations, resistance skills, and psychological inoculation from the original *Hands Off Tobacco!* curriculum (Berman, Guthmann, Crespi, & Liu, 2011).

### The Revised Curriculum

**Figure 1:**
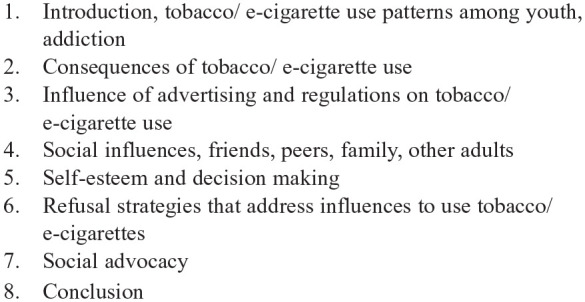
1. Introduction, tobacco/ e-cigarette use patterns among youth, addiction2. Consequences of tobacco/ e-cigarette use3. Influence of advertising and regulations on tobacco/ e-cigarette use4. Social influences, friends, peers, family, other adults5. Self-esteem and decision making6. Refusal strategies that address influences to use tobacco/ e-cigarettes7. Social advocacy8. Conclusion Hands Off Tobacco and E-Cigarettes! Sessions

We moved from grade-level specific lessons (sixth–ninth) to eight topically-organized sessions for use across MS grades (see Figure 1) to accommodate students with varied cognitive development and literacy levels who learn together within grades. We developed interactive and participatory activities for each session and created PowerPoint presentations specific to each session, to guide these activities and classroom discussion. We limited session content to what could be covered in a once weekly, 45-min class period. The revised curriculum feature broad-based social competence, decision-making, peer and other pressures, stereotyping, social isolation, goal setting, self-concept, and other aspects of self-management that are well established in educational programs for DHH youth (Knoors & Marschark, 2014; Sudmand et al., 2020).

To ensure the curriculum was relevant for today’s youth, we updated content related to addiction and health consequences of tobacco use, including information and activities related to e-cigarettes and other novel products (e.g., hookah). To address normative social influences, we highlighted common overestimations about the extent of tobacco and e-cigarette use among adolescents (Sessions 1 and 2). Informational social influences are addressed throughout the curriculum. Misconceptions about harms among e-cigarette users (i.e., “e-cigarettes just contain water vapor” and “vaping is not addictive”) are introduced (Session 2). The role of industry marketing and other social media, as well that of peers, family members, rules and laws, in youths’ choices to experiment with tobacco products is also examined (Sessions 3 and 4). We updated content focused on decision-making (Session 5) and refusal skills (Session 6) and included a social advocacy session in which students create and disseminate anti-tobacco campaigns on campus and via social media (Sessions 7 and 8).

To accompany the classroom materials, we created a comprehensive Teacher's Manual that outlined the content of each session in detail. The manual included a cover page for each session which listed an overarching aim, objectives, necessary materials, and key terms. Teacher’s notes listed important facts to be shared with students, provided instruction about where in the session to play videos, begin discussion, conduct classroom activities, administer quizzes or introduce homework assignments. A vignette featuring DHH students was provided for teachers to read to introduce the content of each session. Discussion questions and multiple-choice response options were also included. To assist teachers in delivering session content, sample language was offered in the form of prompts. We also provided teachers with access to an online repository that included the PowerPoint decks, signed video instructions for take-home activities, digital quizzes, and supplemental materials (e.g., fact sheets) and a student workbook containing handouts, instructions, and space to complete homework assignments.

### Program Delivery

Schools identified opportunities for program delivery in science or physical education classes that serve DHH students only. The five teachers recruited to deliver the curriculum completed a 4-hr, in person, English/ASL training led by two research team members, one fluent in ASL. Trainings discussed program elements, issues in curriculum delivery, strategies for increasing student engagement, and program evaluation elements (student surveys, teacher debriefings).

Although the curriculum was developed for MS students, classes receiving the program included 6th to 12th graders. All students in these classes received the program as part of regular classroom activities whether or not they participated in the research. Packets including an English/Spanish language flyer describing the study and student participation, a consent form for signature, and a postage-paid return envelope were sent to parents/guardians of each student. Assent was obtained from students for whom we received consent, with the assent form printed in English and reviewed in ASL in the classroom by a research team member. Students aged 18 or older provided their own consent.

### Student Surveys

Study participants completed baseline and post-program surveys (see Supplemental Appendix 1). Surveys were administered in the classroom, in ASL, by a trained Deaf ASL-fluent research team member. In keeping with guidance from our Advisory Committee and participating teachers, we sought to keep the survey as brief as possible to ensure that students would be able to complete the survey during one 45-min class period.

Surveys assessed demographic characteristics (Items 1–5) and tobacco product use (Items 6–15). Normative social influences are assessed through items that ask about perceived harms of using tobacco products (Items 17–19), intentions to use tobacco products in the near future and whether or not the respondent would accept an offer of a e-/cigarette from a friend (Items 22–25). Informational social influences are assessed through items that inquire about familial use of tobacco products and e-cigarettes (Item 16) and knowledge about the addictive properties of tobacco products and e-cigarettes (Items 20 and 21). More general tobacco-related knowledge was also assessed (Items 26–35).

Post-program surveys repeated the content of the baseline surveys and included seven additional items aimed to solicit feedback regarding the curriculum (Items 36–39, 41 and 42) and assess prior exposure to tobacco prevention programming (item 40). Survey items were developed by reviewing existing youth tobacco instruments and survey instruments from our prior work among DHH students (National Youth Tobacco Survey (NYTS): Centers for Disease Control and Prevention, 2022); Berman et al., 2000; Berman, Guthmann, Crespi, & Liu, 2011). Students marked responses on paper copies of the surveys. A video of the staff member signing the survey was created for use with students absent for survey administration, and for review by the research team member to ensure consistency across study sites. Students received a US$25 gift card for each completed survey.

Baseline surveys were administered in October to December 2018. The curriculum was delivered at three schools during the 2018–2019 academic year (one mainstream, two Schools for the Deaf) with post-program surveys completed 4 to 6 months later. Due to competing academic priorities, one mainstream school that participated in the baseline survey declined to offer the program and therefore did not participate in other study activities (post-program survey, debriefings).

### Teacher Debriefing

Thirty-min interpreter-assisted debriefings were conducted by the study coordinator, individually, with the five participating teachers (one in person; four by phone via video relay). The study team developed a debriefing guide to structure these conversations, which sought teachers’ feedback regarding program implementation, acceptability, and feasibility, as well as suggestions for next steps (see Supplemental Appendix 2 for Teacher Debriefing Guide). Teachers completed these debriefings within 2 weeks of delivering the program and received US$50 gift cards for their participation in this study activity. Audio recordings of these conversations were reviewed by the study team to identify major themes.

### Data Analysis

We examined participants’ characteristics, knowledge, product use/intention to use at baseline, for the overall sample, and stratified by grade level (MS vs. HS) and school type (Schools for the Deaf vs. mainstream schools). Bivariate comparisons using Fisher exact or two-sample *t* tests were used to evaluate group differences. Bivariate methods also were used to compare participants who completed both baseline and post-program surveys with those who completed only baseline surveys. Changes in knowledge scores (total and individual items) were analyzed for participants who completed both baseline and post-program surveys using McNemar’s or paired *t*-tests surveys to evaluate program effectiveness. Analyses were conducted using SAS (version 9.4, 2013). The *p* value < .05 was considered statistically significant.

## Results

Baseline surveys were completed by 114 students across the four schools, 66% (*n* = 75) from Schools for the Deaf, 56% (*n* = 64) in MS. Table 1 presents student demographic characteristics, tobacco product exposure in the home, and tobacco prevention programming exposure by school type, grade level and overall. Over half of the students (58%, *n* = 66) were male and 54% (*n* = 61) were Latino; 15% were classified as other race/ethnicity, which included students self-identifying with more than one group. Most students identified as Deaf (58%, *n* = 66). With the exception of age, no significant demographic differences were observed by school type or grade level.

**Table 1:** Participant Characteristics

	By school type			By grade level			Total
Characteristic	Schools for the Deaf (n = 75) n (%)	Mainstream schools (n = 39) n (%)	p	Middle school (n = 64) n (%)	High school (n = 50) n (%)	p	(N = 114) n (%)
Age							
11–14 years	56 (76)	22 (56)	.0532	**63 (100)**	**15 (30)**	**<.0001**	78 (69)
15–18 years	18 (24)	17 (44)		**0 (0)**	**35 (70)**		35 (31)
Gender							
Female	32 (43)	15 (39)	.8407	29 (46)	18 (36)	.3383	47 (42)
Male	43 (57)	23 (61)		34 (54)	32 (64)		66 (58)
Grade							
6th–8th	45 (60)	19 (49)	.3203				
9th –12th	30 (40)	20 (51)					
Race/Ethnicity							
Asian/Pacific Islander	8 (11)	4 (10)	.3547	7 (11)	5 (10)	1.000	12 (11)
Black	2 (3)	2 (5)		2 (3)	2 (4)		4 (4)
Latino	36 (48)	25 (64)		34 (53)	27 (54)		61 (54)
White	16 (21)	4 (10)		11 (17)	9 (18)		20 (18)
Other	13 (17)	4 (10)		10 (16)	7 (14)		17 (15)
Self-reported hearing status							
Deaf	46 (61)	20 (53)	.1495	34 (53)	32 (65)	.2942	66 (58)
Hard-of-hearing	29 (39)	16 (42)		28 (44)	17 (35)		45 (40)
Hearing	0 (0)	2 (5)		2 (3)	0 (0)		2 (2)
Exposure to products at home							
Cigarettes	14 (19)	8 (21)	.8072	8 (13)	14 (28)	.0547	22 (19)
E-Cigarettes	**7 (9)**	**10 (26)**	**.0275**	**4 (6)**	**13 (26)**	**.0066**	17 (15)
Cigars/cigarillos	7 (9)	1 (3)	.2606	4 (6)	4 (8)	.7285	8 (7)
Hookah	3 (4)	4 (10)	.2284	**1 (2)**	**6 (12)**	**.0422**	7 (6)
Chewing tobacco	5 (7)	0 (0)	.1635	1 (2)	4 (8)	.1668	5 (4)
Other form of tobacco	**4 (5)**	**9 (23)**	**.0100**	**3 (5)**	**10 (20)**	**.0159**	13 (11)
Any	24 (32)	16 (41)	.4091	**14 (22)**	**26 (52)**	**.0014**	40 (35)
Any prior exposure to tobacco prevention education							
At school, this year	9 (12)	8 (21)	.2711	8 (13)	9 (18)	.4386	17 (15)
At school, before this year	16 (21)	7 (18)	.8072	9 (14)	14 (28)	.0986	23 (20)
At home, this year	3 (4)	2 (5)	1.000	3 (5)	2 (4)	1.000	5 (4)
At home, before this year	6 (8)	7 (18)	.1295	5 (8)	8 (16)	.2366	13 (11)
Any	33 (44)	22 (56)	.2393	**23 (36)**	**32 (64)**	**.0044**	55 (48)

*Note. p* values from Fisher exact tests. Boldface indicates *p* < .05.

Overall, 35% of students reported exposure to tobacco products in the home with significantly greater exposure among HS versus MS students (52% vs. 22%, *p* = .0014) but not varying by school type. Students most commonly reported exposure to cigarettes (19%) and e-cigarettes (15%), with e-cigarette home exposure more common among mainstream than School for the Deaf students (26% vs. 9%, *p* = .0275) and HS than MS students (26% vs. 6%, *p* = .0066). Nearly half of students (48%) reported previously receiving tobacco prevention programming, most commonly at school in a prior year (20%), and more often among HS than MS students (64% vs. 36%, *p* = .0044).

Table 2 presents the students’ baseline tobacco knowledge and use. Approximately one third of students knew that most e-cigarettes have nicotine (35%) and that it is illegal to advertise cigarettes on television (30%); 43% were aware that e-cigarettes can affect brain development. Just over half knew e-cigarettes are prohibited on school grounds (56%) and nicotine is the addictive ingredient in tobacco products (53%), with this knowledge more common among HS than MS students (72% vs. 38%, *p* = .0003). Knowledge did not vary significantly by school type.

**Table 2:** Baseline Knowledge and Tobacco Use (Ever/Current)

	School type			Grade level			Total
Baseline survey item	Schools for the Deaf n = 75 n (%)	Mainstream schools n = 39 n (%)	p	Middle school n = 64 n (%)	High school n = 50 n (%)	p	N = 114 n (%)
Knowledge							
Nicotine is active ingredient in tobacco products (T/F)	41 (55)	19 (49)	**.5601**	**24 (38)**	**36 (72)**	**.0003**	60 (53)
How are harmful chemicals in tobacco carried throughout the body	9 (12)	1 (3)	.1604	6 (9)	4 (8)	1.000	10 (9)
Most e-cigarettes have nicotine in them (T/F)	29 (39)	11 (28)	.3056	19 (30)	21 (42)	.2353	40 (35)
Hookah is a tobacco product (T/F: reverse scored)	36 (48)	16 (41)	.5540	29 (45)	23 (46)	1.000	52 (46)
E-cigarettes can affect brain development	31 (41)	18 (46)	.6917	25 (39)	24 (48)	.3488	49 (43)
Allowed to use e-cigarettes at school but not cigarettes (T/F)	45 (60)	19 (49)	.3203	34 (53)	30 (60)	.5687	64 (56)
It is illegal to advertise cigarettes on TV (T/F)	22 (29)	12 (31)	1.000	15 (23)	19 (38)	.1029	34 (30)
Identify definition of advocate	38 (51)	17 (44)	.5548	27 (42)	28 (56)	.1864	55 (48)
Identify definition of peer pressure	40 (53)	16 (41)	.2403	26 (41)	30 (60)	.0586	56 (49)
Identify example of assertive resistance	30 (40)	11 (28)	.2246	19 (30)	22 (44)	.1216	41 (36)
Total Knowledge Score*, mean + *SD*	4.28 + 2.06	3.59 + 1.89	.0834	**3.50 + 2.07**	**4.74 + 1.74**	**.0009**	4.04 + 2.02
Tobacco product use							
Ever used							
Any product	12 (16)	8 (21)	.6072	**4 (6)**	**16 (32)**	**.0004**	20 (18)
Cigarettes	8 (11)	5 (13)	.7616	**2 (3)**	**11 (22)**	**.0022**	13 (11)
E-cigarettes	7 (9)	7 (18)	.2314	**2 (3)**	**12 (24)**	**.0010**	14 (12)
Cigarettes and E-cigarettes	5 (7)	4 (10)	.4892	**1 (2)**	**8 (16)**	**.0099**	9 (8)
Hookah	5 (7)	2 (5)	1.000	2 (3)	5 (10)	.2372	7 (6)
Cigarillos	2 (3)	0 (0)	.5459	0 (0)	2 (4)	.1902	2 (2)
Current use							
Any product	9 (12)	9 (23)	.1748	**3 (5)**	**15 (30)**	**.0004**	18 (16)
Cigarettes	6 (8)	5 (13)	.5067	3 (5)	8 (16)	.0566	11 (10)
E-Cigarettes	6 (8)	8 (21)	.0718	**1 (2)**	**13 (26)**	**<.0001**	14 (12)
Cigarettes and E-cigarettes	4 (5)	4 (10)	.4423	**1 (2)**	**7 (14)**	**.0206**	8 (7)
Hookah	4 (5)	1 (3)	.6592	**0 (0)**	**5 (10)**	**.0144**	5 (4)
Cigarillos	2 (3)	0 (0)	.5459	0 (0)	2 (4)	.1902	2 (2)

*Note.* Boldface indicates *p* < .05 from Fisher exact tests.

* *p*-values are from two-sample *t* tests.

Overall, 16% of students reported current use of any tobacco product, most commonly e-cigarettes (12%) and cigarettes (10%); 7% reported dual cigarette/e-cigarette use, with use greater among HS than MS students. No statistically significant school-type differences were observed. Few students reported intention to use cigarettes (4%) or e-cigarettes (6%) in the next 30 days; no differences were observed by grade level or school type (data not shown).

### Changes in Product Use and Knowledge

Baseline to post-program comparisons were restricted to students completing surveys at both time points at the three schools where the curriculum was delivered (*n* = 84). Compared with those lost to follow-up (*n* = 30), these students were more likely to be in MS (63% vs. 37%, *p*=.018) and less likely to be current (11% vs. 30%, *p*=.020) or ever tobacco users (11% vs. 37%, *p*=.004). No other demographic differences were observed (data not shown).

Baseline versus post-program comparisons revealed no significant differences in students’ ever/current/intention to use tobacco products (data not shown). Table 3 depicts changes in knowledge (total and by item) overall and stratified by grade level. Changes in knowledge did not differ by school type (data not shown). Among all students, we observed a significant increase in the total knowledge score and in three of the 10 component items. Students’ ability to identify resistance approaches (assertive, aggressive, passive) did not improve. Among MS students, significant improvements occurred in total knowledge and four component items. Among HS students, improvements were limited to two knowledge items; although not statistically significant, we observed an absolute decrease for five items.

**Table 3:** Changes in Knowledge Among Students Who Completed Baseline and Follow-Up Surveys

	By grade level						Total (N = 84)		
	Middle school (n = 54)			High school (n = 30)					
Survey Item	Correct at baseline	Correct at follow-up	P-value change	Correct at baseline	Correct at follow-up	P-value change	Correct at baseline	Correct at follow-up	P-value change
Knowledge items									
Nicotine is the addictive ingredient in tobacco products (T/F)	**24 (45%)**	**35 (66%)**	**.0076**	22 (71%)	18 (58%)	.1025	46 (55%)	53 (63%)	.1444
How are harmful chemicals in tobacco carried throughout the body	**5 (9%)**	**17 (32%)**	**.0047**	**2 (6%)**	**10 (32%)**	**.0114**	**7 (8%)**	**27 (32%)**	**.0002**
Most e-cigarettes have nicotine in them (T/F)	**16 (30%)**	**27 (51%)**	**.0116**	**12 (39%)**	**21 (68%)**	**.0201**	**28 (33%)**	**48 (57%)**	**.0006**
Hookah is a tobacco plant (reverse scored)	25 (47%)	30 (57%)	.2513	13 (42%)	13 (42%)	1.000	38 (45%)	43 (51%)	.3841
E-cigarettes affect brain development	**21 (40%)**	**34 (64%)**	**.0158**	16 (52%)	21 (68%)	0.0588	**37 (44%)**	**55 (65%)**	**.0027**
Allowed to use e-cigarettes at school but not cigarettes (T/F)	27 (51%)	34 (64%)	.0896	18 (58%)	18 (58%)	1.000	45 (54%)	52 (62%)	.1615
Advertising of cigarettes is allowed on TV (T/F)	12 (23%)	16 (30%)	.2482	12 (39%)	6 (19%)	.0833	24 (29%)	22 (26%)	.6831
Identify definition of advocate	24 (45%)	28 (53%)	.3458	19 (61%)	16 (52%)	.4386	43 (51%)	44 (52%)	.8618
Identify definition of peer pressure	24 (45%)	24 (45%)	1.000	18 (58%)	17 (55%)	.7389	42 (50%)	41 (49%)	.8415
Identify example of assertive resistance	17 (32%)	17 (32%)	1.000	16 (52%)	13 (42%)	.4386	33 (39%)	30 (36%)	.6121
Total Knowledge Score (*SD*)*	**3.68 (2.14)**	**4.94 (2.58)**	**<.0001**	4.77 (1.69)	4.94 (2.31)	.6646	**4.08 (2.04)**	**4.94 (2.47)**	**.0003**

*Note.* Boldface indicates *p* < .05 from McNemar’s tests.

* *p* values are from paired *t* tests.

### Student Feedback

On post-program surveys, a majority of students indicated that they became “really involved” in curriculum topics and activities (65%), expected to remember what was taught 1 year later (73%), believed they would be able to use the information provided (62%), and would recommend the program to other DHH students their age (64%). No significant differences in these items were observed by school type or grade level, except that a greater proportion of HS than MS students reported being likely to remember what they learned in a year (86% vs. 67%, *p* = .0472). Eight students wrote comments describing group discussions and activities as their favorite part of the program. A smaller number reported that the amount of required writing was challenging (*n* = 4), the program was “boring” (*n* = 3) or it was “really long” (*n* = 1; data not shown).

### Teacher Feedback

Feedback was obtained through teacher debriefings. All five teachers indicated that students were engaged and eager to learn about e-cigarette and tobacco products; the curriculum fills an important educational need for DHH students; and the greatest challenge in curriculum use was that sessions took longer to deliver than anticipated, largely due to students’ widely varied educational and literacy levels and the associated need to make appropriate modifications and revisions. All of these teachers (*n* = 5) strongly urged greater inclusion of multimedia content, for example, videos to illustrate vocabulary and resistance strategies, emphasizing that videos should be in ASL and feature DHH actors to promote students’ identification with the program. One teacher also noted that English language captioning and voiceover are needed to ensure access for hearing students, important in mainstream settings. Two of these educators also recommended customized student workbooks for varied reading levels, supplemental information regarding concepts introduced in class, sentence starters for exercises requiring writing, examples of completed activities, and including digital and social-media e-cigarette marketing examples. Another teacher suggested allowing students to sign answers to in-class and homework questions using video (“Voice Thread”) or to draw rather than write responses. Reflecting their view that DHH youth often have especially strong bonds with their peers, several teachers (*n* = 3) recommended greater emphasis on peer pressure.

## Discussion

Significant barriers to receipt of health messages and results from this pilot work underscore the need for culturally and linguistically tailored school-based tobacco use prevention education for DHH youth. Despite the small number of students in this exploratory, formative work, because of the limited research in this area, we decided to examine the characteristics, practices, and baseline to post-program changes in knowledge and practices among participants overall and by school type and grade level. In doing so, we found evidence of tobacco use, low levels of knowledge, and considerable exposure to product use at home among study participants. Although we recognize that knowledge is insufficient to change behavior, we were pleased to see increased knowledge associated with program receipt, as it is, nonetheless, an important component of behavior.

This study expands on our prior tobacco work with DHH youth (Berman et al., 2007; Berman, Guthmann, Crespi & Liu, 2011; Berman, Guthmann, Liu & Streja, 2011) with the inclusion of students in mainstream educational settings. This represents an important step forward given DHH students increasingly learn in these settings rather than in Schools for the Deaf. Whereas studies that include a focus on students in both settings tend to highlight differences in the educational and social experiences of students in these two settings (e.g., Andersson & Lyngback, 2022; Angelides & Aravi, 2007), we found study participants in Schools for the Deaf and mainstream settings were similar in tobacco knowledge and behavior. In both settings, grade level differences were observed, including greater e-cigarette use among HS than MS students. Although it may be that DHH students begin product use later than their hearing peers, who most often begin in MS (DHHS, 2012), given the cross-sectional nature of this study, we are unable to determine the age of uptake in this population, which is a limitation of this study.

Although students reported some prior tobacco prevention education, including school-based programming, conversations with students and teachers suggest that content was limited, often nested as a brief school-wide component in a broader program. There were no reports of the type of in-depth classroom programming widely accepted as the gold standard in school-based tobacco prevention and that we sought to provide.

Student and faculty feedback suggest that what we have already developed, to our knowledge the only school-based tobacco prevention curriculum specifically tailored for DHH students, represents a worthwhile foundation for much-needed programming. It is encouraging that program content organized topically into eight sessions was well received and seen as appropriate for both MS and HS students. Nonetheless, we remain cognizant of the challenges associated with developing uniform materials given students’ highly varied reading levels and language deprivation. We expect that the inclusion of extensive video content in future programming will help to address this issue. We will continue including students and faculty from Schools for the Deaf and mainstream schools in future research and remain alert to possible differences in programming requirements in these settings. However, our preliminary work suggests that it is reasonable to move forward with one tailored curriculum and that the program we are developing would likely be appropriate for use in both school environments.

We also learned, from the limited improvements in knowledge achieved and the comments of students and teachers, that additional changes are needed to make the program more engaging, maximize its impact, and increase the likelihood of implementation within already full school curricula. While retaining important elements of school-based tobacco prevention programming, sessions should be further streamlined and more tightly focused, with content crafted at a low literacy level to facilitate use with students at diverse reading and educational levels. Aspects of DHH youth culture, for example, the prominence of peer influence (Millen et al., 2019; Terlektsi et al., 2020), should be emphasized. More extensive use of visual images and digital content, beyond the scope of this pilot study given our limited resources, was described as crucial. Although use of signing, ASL, was considered critical, so too was including English voice over and captioning to accommodate hearing youth who may be exposed to the program in mainstream settings alongside their DHH classmates. Finally, teachers commented that programming needed to be kept up-to-date and comprehensive regarding evolving marketing practices and products to retain student interest. With this in mind, while recognizing that it is outside the scope of this project, these educators noted that marijuana use comes up frequently in classroom discussions and other conversations with students and is a concern that calls for programming attention.

We will build on what we have learned in this pilot study regarding additional needed curriculum changes and concerning data collection among DHH youth. For example, our inability to detect improved understanding of various resistance strategies may reflect students’ challenges in learning complex vocabulary terms rather than failure to acquire the necessary resistance skills. In future work, ASL videos could be used to teach complex terminology with live or video-recorded demonstrations used to assess mastery of these skills. Given DHH students varied educational levels, observational methods may be better suited to evaluation. In light of the relatively small number of students in this pilot study that was focused primarily on MS students, we will next conduct a larger trial to test the program’s effectiveness, specifically including HS and MS students. Our results illustrate the importance of ensuring curriculum effectiveness for DHH HS students, among whom we observed tobacco use, gaps in knowledge, and given they may learn alongside MS students. In future work, we will also seek to ensure greater student participation at all data collection time points. Unfortunately, the loss of students at follow-up in this study inhibited our ability to examine program effects among students who reported tobacco use, a limitation of this study. While focusing on linguistic and cultural tailoring for DHH students, we will also ensure access for hearing youth; examine issues of intersectionality such as Latino/a/x ethnicity and hearing status given increased e-cigarette use observed among Latino/a/x youth at the population level (Park-Lee et al., 2021); and seek opportunities for additional research to develop and implement strategies for national curriculum dissemination. Additional limitations of this work include the small number of participating schools, teachers, and students and that this pilot work was conducted within only one state. We recognize that exposure to tobacco products and marketing, use patterns, and student knowledge and beliefs may vary in other parts of the country. Despite these shortcomings, this study presents many important lessons for educators and demonstrates the promise of our modified tobacco prevention curriculum for the education of DHH youth in varied educational settings.

## Implications for Practice

Gaps in tobacco-related knowledge, use, exposure, and limited receipt of comprehensive classroom programming underscore the need for increased intervention for DHH youth. The MS/HS differences we found, along with teacher comments, suggest that gaps in knowledge were particularly prevalent among MS students and that uptake and use may occur later in this population than among hearing youth (Park-Lee et al., 2021; Titus et al., 2008). Given this, it is important for educators and school administers to recognize the need for tailored, school-based tobacco prevention education for DHH students at both the HS and MS levels. In keeping with the feedback we received from students and teachers, we encourage educators who will deliver any type of prevention programming for DHH youth to incorporate group activities and visual media, including videos, that feature DHH youth to the extent possible. We also recommend that careful attention is paid to estimate time needed for program delivery. We learned that additional time is often required to navigate the varied educational and literacy levels of students within classrooms when serving those who are DHH or have other special needs. In addition, feedback from teachers underscores the value of programs that utilize multiple languages (e.g., English and ASL) to ensure accessibility for all students. This is particularly important given the trend toward mainstream education among DHH youth, and a promising practice given the few differences observed between students attending Schools for the Deaf and mainstream schools. A program that allows DHH youth to learn side-by-side with their hearing peers, utilizing the same materials and minimal external supports, holds the promise of enriching the education of all students, is reflective of a commitment to educational and health equity and can contribute to meeting the needs of this traditionally underserved population.

## Supplemental Material

sj-docx-1-hpp-10.1177_15248399221151180 – Supplemental material for Developing and Evaluating a School-Based Tobacco and E-Cigarette Prevention Program for Deaf and Hard-of-Hearing Youth Click here for additional data file. Supplemental material, sj-docx-1-hpp-10.1177_15248399221151180 for Developing and Evaluating a School-Based Tobacco and E-Cigarette Prevention Program for Deaf and Hard-of-Hearing Youth by Alison K. Herrmann, Burton Cowgill, Debra Guthmann, Jessica Richardson, L. Cindy Chang, Catherine M. Crespi, Everett Glenn, Michael McKee and Barbara Berman in Health Promotion Practice
