# Supplementary material for: Developing and Evaluating a School-Based Tobacco and E-Cigarette Prevention Program for Deaf and Hard-of-Hearing Youth
Source: Health Promot Pract. 2023 Feb 9;25(1):65–76. doi: 10.1177/15248399221151180 (PMC10768334; doi:10.1177/15248399221151180)
Supplement: sj-docx-1-hpp-10.1177_15248399221151180 – Supplemental material for Developing and Evaluating a School-Based Tobacco and E-Cigarette Prevention Program for Deaf and Hard-of-Hearing Youth [file sj-docx-1-hpp-10.1177_15248399221151180.docx]

**Appendix 1**

**Baseline Survey (Items 1-35)**

**Post-Program Survey (Items 1-42)**

1. What is your age? ________________years old
2. What is your gender?

____Male ____Female ____Other ____Prefer not to answer

1. How would you describe yourself? (*MARK ONE OR MORE RESPONSES*)

____American Indian or Alaska Native

____Asian

____Black or African American

____Filipino

____Latino or Hispanic

____Native Hawaiian or Other Asian Pacific Islander

____White

____Other (*please specify*) ______________________________

1. What is your grade level? (*MARK ONE ANSWER*)

___ 6^th^ grade

___ 7^th^ grade

___ 8^th^ grade

___ 9^th^ grade

1. How would you describe yourself? (*MARK ONE ANSWER*)

___ Deaf

___ Hard-of-Hearing

___ Hearing

1. Have you ever tried cigarette smoking, even one or two puffs?

___ Yes

___ No

1. During the past 30 days, on how many days did you smoke cigarettes?

___ 0 days

___ 1 or 2 days

___ 3 to 5 days

___ 6 to 9 days

___ 10 to 19 days

___ 20 to 29 days

___ All 30 days

1. Have you ever tried chewing tobacco, snuff, or dip, even just a small amount?

___ Yes

___ No

1. During the past 30 days, on how many days did you use chewing tobacco, snuff, or dip?

___ 0 days

___ 1 or 2 days

___ 3 to 5 days

___ 6 to 9 days

___ 10 to 19 days

___ 20 to 29 days

___ All 30 days

1. Have you ever tried an electronic or e-cigarette (vape), even one or two puffs?

___ Yes

___ No

1. During the past 30 days, on how many days did you use an electronic or e-cigarette?

___ 0 days

___ 1 or 2 days

___ 3 to 5 days

___ 6 to 9 days

___ 10 to 19 days

___ 20 to 29 days

___ All 30 days

1. Have you ever tried hookah, even one or two puffs?

___ Yes

___ No

1. During the past 30 days, on how many days did you use hookah?

___ 0 days

___ 1 or 2 days

___ 3 to 5 days

___ 6 to 9 days

___ 10 to 19 days

___ 20 to 29 days

___ All 30 days

1. Have you ever tried cigarillos, even one or two puffs?

___ Yes

___ No

1. During the past 30 days, on how many days did you use cigarillos?

___ 0 days

___ 1 or 2 days

___ 3 to 5 days

___ 6 to 9 days

___ 10 to 19 days

___ 20 to 29 days

___ All 30 days

1. Does anyone who lives with you now…? (MARK ALL THAT APPLY)

___Smoke cigarettes

___Smoke cigars

___Smoke cigarillos

___Use a hookah

___Use chewing tobacco

___Use e-cigarettes

___Use any other form of tobacco

___No one who lives with me now uses any form of tobacco

1. Is using e-cigarettes less harmful, about the same, or more harmful than smoking regular cigarettes?

___Less harmful

___About the same

___More harmful

1. How much do you think people harm themselves when they use e-cigarettes?

___No harm

___A little harm

___Some harm

___A lot of harm

1. How much do you think people harm themselves when they use regular cigarettes?

___No harm

___A little harm

___Some harm

___A lot of harm

1. How likely is someone to become addicted to e-cigarettes, meaning it would be hard for them to stop using e-cigarettes?

___Very unlikely

___Somewhat unlikely

___Neither likely nor unlikely

___Somewhat likely

___Very likely

1. How likely is someone to become addicted to regular cigarettes, meaning it would be hard for them to stop using cigarettes?

___Very unlikely

___Somewhat unlikely

___Neither likely nor unlikely

___Somewhat likely

___Very likely

1. Do you think that you will use an e-cigarette in the next 30 days?

___Definitely yes

___Probably yes

___Probably no

___Definitely no

1. If one of your best friends offered you an e-cigarette, would you use it?

___Definitely yes

___Probably yes

___Probably no

___Definitely no

1. Do you think that you will try a regular cigarette in the next 30 days?

___Definitely yes

___Probably yes

___Probably no

___Definitely no

1. If one of your best friends offered you a regular cigarette, would you use it?

___Definitely yes

___Probably yes

___Probably no

___Definitely no

*To the best of your ability, provide ONE correct answer to questions 26-35.*

1. Nicotine is the addictive ingredient in tobacco products.

___True

___False

___Not sure

1. The harmful chemicals in tobacco products are carried throughout your body by…

___Your lungs

___Your blood

___Your heart

1. Most e-cigarettes have nicotine in them.

___True

___False

___Not sure

1. You are allowed to use e-cigarettes at school, but not cigarettes.

___True

___False

___Not sure

1. Someone who speaks up in support for or against something they believe in is …

___A writer

___An advocate

___A salesman

1. Define peer pressure.

___To be influenced by adults

___To be influenced by someone your own age

___To be influenced by advertising

1. Hookah is a tobacco product.

___True

___False

___Not sure

1. You can advertise cigarettes on television.

___True

___False

___Not sure

1. Which of the following statements is TRUE about e-cigarettes?

___ E-cigarettes produce water vapor

___ Using e-cigarettes can affect brain development

___ Fruit flavorings used in e-cigarettes makes them safe

1. Assertive resistance means which of the following?

___ Using a confrontational tone to make your point

___ Using a clear and confident tone to make your point

___ Ignoring the situation instead of making your point

*Earlier this year, your class participated in an e-cigarette and tobacco education curriculum called Hands Off Tobacco and E-Cigarettes! The next set of questions asks your opinions about the program. Take a minute to think about all eight Hands Off Tobacco and E-Cigarettes! sessions. Think about the topics and activities you did each time you met. Try to form a general opinion about the program. Now answer the following questions.*

1. Would you recommend that other Deaf and Hard-of-Hearing students your age be given the Hands Off Tobacco and E-Cigarettes! program?

___ Yes, most of it

___ Maybe, some of it

___ No, not really

1. Do you feel that you will be able to use the information taught in Hands Off Tobacco and E-Cigarettes!?

___ Yes, most of it

___ Maybe, some of it

___ No, not really

1. A year from now, will you remember what was taught in Hands Off Tobacco and E-Cigarettes!?

___ Yes, most of it

___ Maybe, some of it

___ No, not really

1. Did you get really involved with the topics and activities in Hands Off Tobacco and E-Cigarettes!?

___ Yes, most of it

___ Maybe, some of it

___ No, not really

1. Have you participated in any other tobacco and e-cigarette prevention program other than Hands Off Tobacco and E-Cigarettes! (*CHECK ALL THAT APPLY*)

___Yes, this year at school

___ Yes, before this year at school

___ Yes, this year outside of school

___ Yes, before this year outside of school

___ No, never

1. What was the ONE thing you liked the MOST about Hands Off Tobacco and E-Cigarettes!?

**___________________________________________________________________________________**

1. What was the ONE thing you liked the LEAST about Hands Off Tobacco and E-Cigarettes!?

**_____________________________________________________________________________________**

**Appendix 2
Teacher Debriefing Guide**

Feedback regarding students who received the program

1. Can you tell me more about the students you taught this program to? (number of classes, number of students, type of students (e.g., reading abilities))
2. Do you think your students liked participating in Hands Off Tobacco and E-Cigarettes!? What, if anything, did they mention to you about HOT!? Likes or dislikes?
3. Which activities were the most well received by your students? Which were least well received?
4. What would you suggest to make the curriculum and lessons more appropriate for DHH students?

Feedback about teachers’ experience delivering the program

1. How long did it take you to teach each session, on average? Do you think the sessions were too long, too short, or just about the right length?
2. What was the most challenging part of teaching this curriculum? What can we do to help make it easier/simpler?
3. Let’s take a look at the evaluations you filled out after each session. Were there any activities you chose to skip or felt like you needed to skip? Were there any sessions you needed to adapt to meet the needs of your students?
4. Did you find the layout of the printed teacher’s manual to be easy or difficult to follow? What changes would you suggest?
5. Was the online resource folder helpful? What changes would you suggest?
6. Did the students utilize the workbooks? What changes would you suggest?

Recommendations for future steps

1. Which components of the curriculum would you prioritize for video content?
2. Would you recommend HOT! to other programs serving DHH students? Why or why not?

Do you have any final comments or recommendations?
